# Children plan manual actions similarly in structured tasks and in free play

**DOI:** 10.1016/j.jecp.2024.106124

**Published:** 2024-11-22

**Authors:** Sara Golmakani, Brianna E Kaplan, Karen E Adolph, Ori Ossmy

**Affiliations:** a Centre for Brain and Cognitive Development and School of Psychological Sciences, Birkbeck, University of London, London WC1E 7JL, UK; b Department of Psychology, The University of Texas at Austin, Austin, TX 78712, USA; c Department of Psychology, New York University, New York, NY 10003, USA

**Keywords:** Object manipulation, Manual actions, Planning, Tool use, Multi-step planning, Free play, Children, Motor development

## Abstract

Visually guided planning is fundamental for manual actions on objects. Multi-step planning—when only the requirements for the initial action are directly visible in the scene—necessitates initial visual guidance to optimize the subsequent actions. We found that 3- to 5-year-old children (*n* = 23) who exhibited visually guided, multi-step planning in a structured tool-use task (hammering down a peg) also demonstrated visually guided planning during unstructured free play while interlocking Duplo bricks and Squigz pieces. Children who exhibited visually guided planning in the hammering task also spent more time looking at the to-be-grasped free-play object and at their construction during reach and transport compared with children who did not demonstrate multi-step planning in the hammering task. Moreover, visually guided planning in the Duplo and Squigz tasks was positively correlated, indicating that planning generalizes across contexts. Findings show that visually guided planning in young children generalizes across different manual actions on objects, including structured tool use and unstructured free play.

## Introduction

### Planning to plan

To meet the demands of daily life, motor actions must be adapted to changes in the environment. Actions cannot be repeated in exactly the same way, even when performed on the same object with the same end goal, because the circumstances of everyday tasks are always changing. Think of children’s everyday manual actions like fitting shapes into a shape-sorter toy or a button into its button-hole, using a spoon to eat or a hammer to pound a peg, stacking blocks to build a tower, and arranging magnet pieces to construct a design. Each action requires continual adaptations to meet the task demands—changing the reach trajectory to grasp the object at varying distances, altering the grip configuration to grasp the object at different orientations, modifying the subsequent arm and hand movements to transport the object to different locations, and so on.

Such adaptation in manual actions involves *planning* so that the sequence of motor behaviors is organized prospectively (Adolph & Robinson, 2015; Keen, 2011). Planning always entails gathering perceptual information ahead of time, typically visual information about the objects and environment. Visually guided action planning develops concurrently with improvements in manual skills so that children’s actions become faster, more accurate, and more efficient (Lockman et al., 2018). In particular, children must learn to put their eyes on the most informative parts of the scene so as to guide their subsequent manual actions—a sort of “planning to plan” (Ossmy et al., 2020, 2022). For example, when fitting shapes into a shape-sorter toy, adults look at the target shape before they reach for it and look at the appropriate aperture before and during transport; thus, the shape slides quickly into the shape-sorter in one smooth motion. In contrast, young children do not point their gaze at the target shape until after they begin to reach and do not fixate the target aperture until after they begin to transport the object; as a consequence, children must correct the location and orientation of the object after it arrives at the shape-sorter box, and their object fitting is slower, less accurate, and less efficient compared with adults (Ossmy et al., 2020). Likewise, variations in object size and shape lead to failures in children’s planning and execution while fitting shapes into apertures (Ornkloo & von Hofsten, 2007; Shutts et al., 2009).

**Fig. 1.**
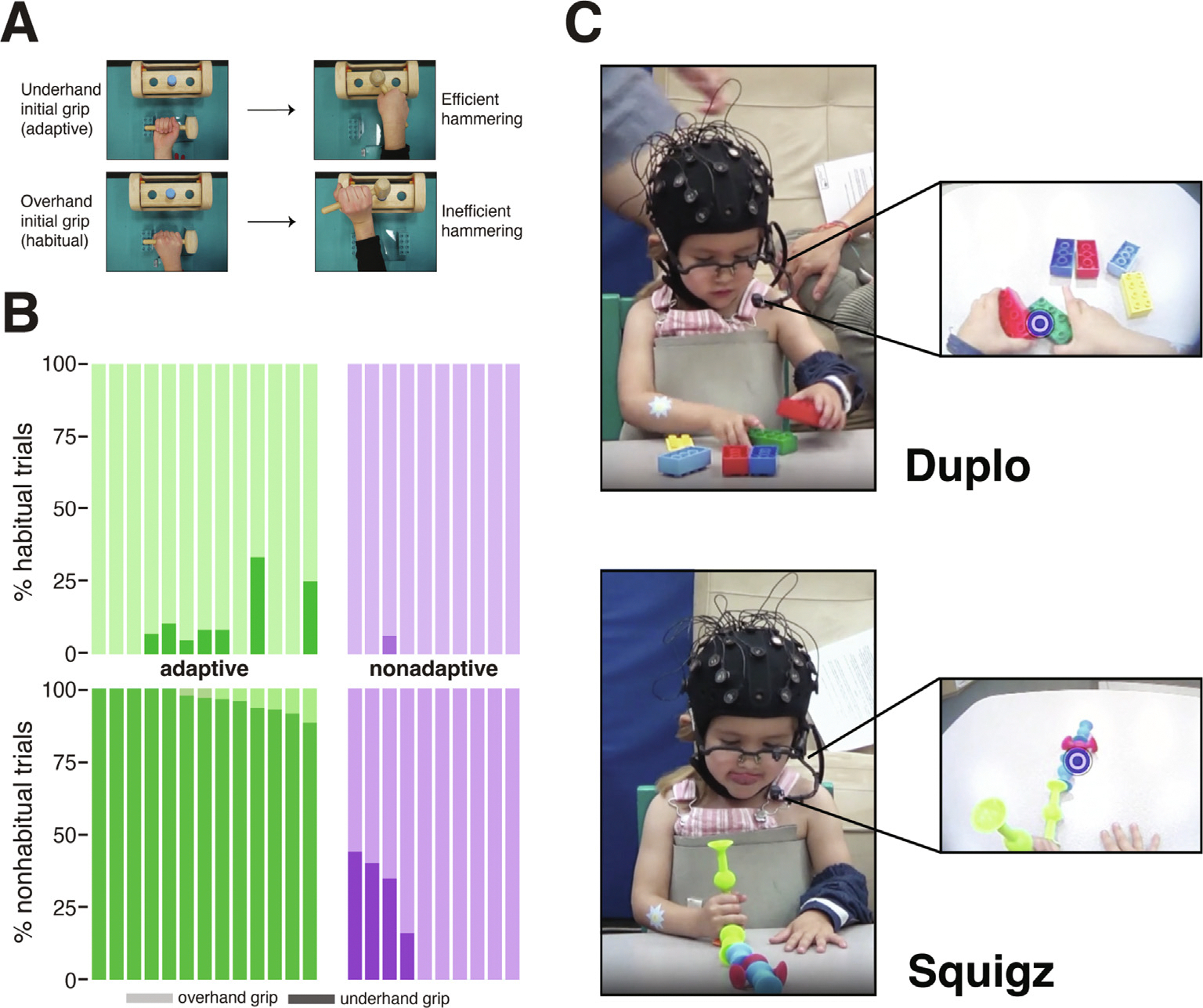
Structured hammering task and unstructured free-play construction tasks. (A) Hammering task when handle of hammer points away from dominant hand (“nonhabitual” trials). Initial grips and subsequent hand position for hammering are shown. Top: Nonhabitual underhand/radial grip leads to an efficient comfortable position for pounding the peg. Bottom: Habitual overhand/ulnar grip leads to an awkward position for pounding the peg. (B) “Adaptive” and “nonadaptive” children in the hammering task grouped by their grips on nonhabitual trials. Each pair of vertical bars represents a single child in each condition, and bars are ordered according to the child’s percentage of nonhabitual grasps on nonhabitual trials. On habitual trials (top panel; hammer handle pointed toward dominant hand), all children used a habitual overhand grip (light bars). On nonhabitual trials (bottom panel; hammer handle pointed toward the nondominant hand), 13 adaptive children used underhand grips (>82.5% of nonhabitual trials) and 10 nonadaptive children continued to use the habitual overhand grip or used both overhand and underhand grips (from Ossmy et al., 2022). (C) Free-play tasks. Duplo bricks (top panel) and Squigz pieces (bottom panel) are shown. Inset frames show the view from the eye-tracker (blue dot indicates gaze location).

Multi-step planning is even more complex because the initial action must take subsequent actions into account (Rosenbaum et al., 1990, 2012). The complexity arises because the constraints of the second action, which are not immediately perceptible, influence the initial action, which is directly accessible in the scene. For manual actions, sometimes a nonhabitual or initially awkward grip facilitates an efficient, comfortable end-state position for a subsequent manual action (Rosenbaum et al., 1990). For example, if the handle of a spoon or hammer points away from the reaching hand, then a nonhabitual, underhand grip is needed to position the hand to bring food to the mouth or to hammer a peg (e.g., Fig. 1A). Infants and young children often fail to plan multiple steps in advance. Instead, they rely on their habitual overhand grip so that food falls off the spoon during transport or their hand is ill-positioned to hammer the peg (Comalli et al., 2016; Keen, 2011; McCarty et al., 1999; Wunsch et al., 2013).

As with single-step planning, multi-step planning develops as children learn to gather the relevant visual information, integrate it into an action plan, and then execute the movements. Simultaneous recording of visual fixations (via head-mounted eye-tracking), neural activation (based on electroencephalography), arm movements (using high-speed motion tracking), and grip configuration (based on video) showed a sequenced real-time planning cascade of perceptual, neural, and motoric events as children and adults grasped a hammer to pound down a peg (Ossmy et al., 2022). When the handle pointed toward their dominant hand, participants of all ages used a habitual overhand grip. But when the handle pointed away from their dominant hand, every adult—but only some of the children—used a nonhabitual underhand grip (Fig. 1A and 1B). Adults and the subset of children in the “adaptive” group fixated the hammer immediately so that they had time to integrate the visual information into the action plan and to choose the adaptive underhand grip instead of the habitual overhand one. In contrast, children in the “nonadaptive” group fixated the hammer too late and thus used the habitual grip that led to inefficient hammering or repositioning and regrasping the hammer (Ossmy et al., 2022).

### Visually guided planning and task constraints

Prior work on the development of visually guided planning of manual actions, however, is limited to structured tasks with a clear goal and a singular method of achievement like using a spoon to bring food to the mouth, pounding down a peg with a hammer, or fitting objects into a shape-sorter toy (Comalli et al., 2016; Keen, 2011; McCarty et al., 1999; Ossmy et al., 2020; Ossmy et al., 2022). But real-world situations often allow for multiple ways to accomplish daily activities. Adults’ gaze strategies differ between natural activities and experimenter-instructed tasks (Lappi, 2015). Perhaps gaze strategies likewise differ when the task constraints are self-determined by the children rather than mandated with explicit instructions from an experimenter. If visually guided planning is a domain-general ability that children increasingly incorporate into their daily lives across development, the same children who plan ahead in one manual task should plan ahead in other manual tasks regardless of the task demands. Free play with blocks and other construction toys presents an ideal context to test the generalization of visually guided planning of manual actions because it avoids the rigid constraints of externally imposed instructions but possesses its own inherent rules and goals (Chu & Schulz, 2020).

Here, we observed children’s visual fixations and manual actions on objects during free play to test whether visually guided planning generalizes from structured manual tasks to unstructured free play. We analyzed free-play data from “filler” tasks with the same 3- to 5-year-olds who hammered down pegs in Ossmy et al. (2022). Between blocks of the hammering task, children played freely with two types of construction toys: Duplo interlocking bricks and Squigz suction-cup pieces (Fig. 1C). Both toys can be used for construction but vary in their visual and tactile properties and the dexterity required for construction. In addition, Duplo bricks are familiar to most children, but Squigz pieces are not. We identified each attempt to connect a new Duplo brick or Squigz piece to the existing structure and noted the times when the reach began and the transport ended. We then examined where children looked during reach and transport to understand the real-time planning cascade during free play.

Our first aim was to test whether visually-guided, multi-step planning in a structured tool-use task generalizes to visually-guided planning in unstructured free play with construction toys. The analyses from Ossmy et al. (2022) divided children into a group who showed adaptive, efficient multi-step planning (termed “adaptive”; Fig. 1B, left panel) and children who did not (“nonadaptive”; Fig. 1B right panel). We predicted that the adaptive children from the structured tool-use task would show earlier information gathering (looking more at the target object and construction during reach and transport) than the nonadaptive children when playing freely with objects. We also predicted better planning with Duplo bricks, a toy all children had experience with, than with Squigz pieces, a novel toy.

Our second aim was to test whether children’s visually-guided planning generalizes across unstructured free play with different toys. We predicted that children who showed high levels of looking during reach and transport with Duplo bricks would also show high levels of looking during reach and transport with Squigz pieces.

## Method

With parents’ permission, videos and demographic data are shared with authorized investigators in the Databrary web-based library. The hammering and free-play videos are shared at databrary.org/volume/434. Illustrative video examples of hammering, Datavyu video annotation spreadsheets, Datavyu scripts, processed data, and analysis scripts are publicly shared at databrary.org/volume/434. Illustrative video examples of free play with Duplo bricks and Squigz pieces, Datavyu video annotation spreadsheets, Datavyu scripts, processed data, and analysis scripts are publicly shared at databrary.org/volume/434.

### Participants

Children were recruited from the New York City area. The final sample included 23 children (*M*_age_ = 3.94 years; 14 girls and 9 boys). Due to malfunctioning of the eye-tracker (*n* = 5) or insufficient data (fewer than four attempts to interlock Duplo bricks or Squigz pieces, *n* = 3, or not looking at the toy, *n* = 1), we did not analyze data from an additional 9 children in the original sample reported in Ossmy (2022). All children had normal or corrected-to-normal vision, and none had cognitive deficits or neurological problems. All reported prior experience with Duplo or Lego bricks, but none reported experience with Squigz pieces. Children received a robot toy, photo magnet, and tote bag for their participation. Based on the analyses from Ossmy (2022), 13 children were considered adaptive (*M*_age_ = 4.16 years; 8 girls and 5 boys) and 10 were considered nonadaptive (*M*_age_ = 3.67 years; 6 girls and 4 boys). The adaptive children were older than the nonadaptive children, *t*(20) = −3.56, *p* = .002.

### Procedure

Between blocks of hammering trials, children engaged in one 2.5-min free-play trial with Duplo bricks and one 2.5-min free-play trial with Squigz pieces. Children sat at a child-sized table across from or next to an experimenter. Caregivers sat behind their children, filling out a questionnaire.

The Duplo trial involved six differently colored bricks with the same rectangular shape and size (1. 9 × 3.1 × 6.3 cm) (Fig. 1C, top panel). Duplos interlock by fitting the studs on the top of one brick into the spaces on the bottom of another brick. The Squiqz trial involved six pieces with different colors, sizes (2.5–9 cm in length), and numbers of suction cups (1–3) (Fig. 1C, bottom panel). Squigz pieces interlock by pressing the suction cup of one piece to the suction cup of another piece. The Duplo bricks were always offered to the children between the first and second blocks of hammering, and Squigz pieces were offered between the second and third blocks of hammering.

Trials began when the experimenter spread the objects out on the table within arm’s reach of children and asked, “What can you do with these?” The experimenter busied herself with paperwork so that children would play on their own, but occasionally she offered encouragement if children bid for attention (e.g., “Wow, that’s so cool, what else can you do?”). Children were free to choose what they did with the toys, including how many times they assembled and disassembled the pieces.

Two fixed cameras recorded children’s hands from top and side views and were mixed online onto a single video. Children’s gaze was recorded with a head-mounted eye-tracker and synced offline with the third-person cameras. Details of the head-mounted eye-tracker and synchronization can be found in Ossmy et al. (2022, p. 11) and on Databrary (databrary.org/volume/434).

### Video coding

A primary coder annotated videos frame by frame using Datavyu software (datavyu.org) that time-locks user-defined events with their location in the video. The coder identified each time children attempted to connect a Duplo brick or Squigz piece (i.e., target object) to an existing construction. The coder marked the onset of reaching when children moved their hand toward the target object and the offset of reaching when the object was lifted from the table. The onset of transport began at the offset of reaching, and the offset of transport was when the object touched the construction.

Using the gaze cursor in the eye-tracking video, the coder also identified the duration of each fixation on the target object or construction versus other objects on the table. We did not separate looking at the target object from looking at the construction because the two were located close to each other in children’s field of view and the construction occasionally blocked the view of the object. Given the error in the eye-tracking technology, we could not reliably distinguish whether children were looking at the object or the construction.

Moreover, because analyses focused on when children looked at the target object or construction, we only coded attempts when children brought a target object to a construction of two or more pieces. Bringing together only two pieces did not count as an attempt because we could not determine which object was the target and which was the construction.

To ensure interobserver reliability, a second coder independently scored 100% of the manual actions and 25% of the visual actions (a smaller percentage because coding visual fixations was more laborious). Interobserver agreement for manual actions was 97.12%, *kappa* = .84, *p* < .01. Interobserver agreement for accumulated durations of fixations to the target object and construction was high, *r*s(20) > .89, *p*s < .001, and the two coders showed exact agreement for at least 96.23% of video frames for the fixated regions of interest, *kappas* > .85, *p*s < .001.

### Data analyses

To account for relatively small sample sizes, we considered each attempt to interlock as its own trial. To compare visually guided planning in free play between adaptive and nonadaptive children, we conducted linear mixed-effect models on the proportion of time children looked at the object and construction during reach and transport separately and together. We conducted separate models for each toy, with child as a random effect and planning status (adaptive or nonadaptive) and age as fixed effects. Because adaptive and nonadaptive children differed in age, including age in the models increased model fit (evidenced by likelihood ratio tests). To test for the main effect of planning status, each model was compared with a null model (with only child and age included) with a likelihood ratio test. Preliminary analyses showed no effect of children’s sex (*p* > .09), so it was collapsed in subsequent analyses. To compare visually guided planning across free play tasks, we correlated children’s looking during reach and transport across Duplo and Squigz trials.

## Results

Children performed a total of 288 attempts to interlock Duplo bricks and 259 attempts to interlock Squigz pieces (*M*s = 8.96 and 7.39 attempts per child, respectively). The numbers of attempts did not differ across tasks, *t*(22) = 1.03, *p* = .32, or between adaptive and nonadaptive children in either task, *t*s < 1.73, *p*s > .10. Because each attempt varied in duration, we transformed looking times into the percentage of the reach and transport phase in which children looked at the target object or construction.

**Fig. 2.**
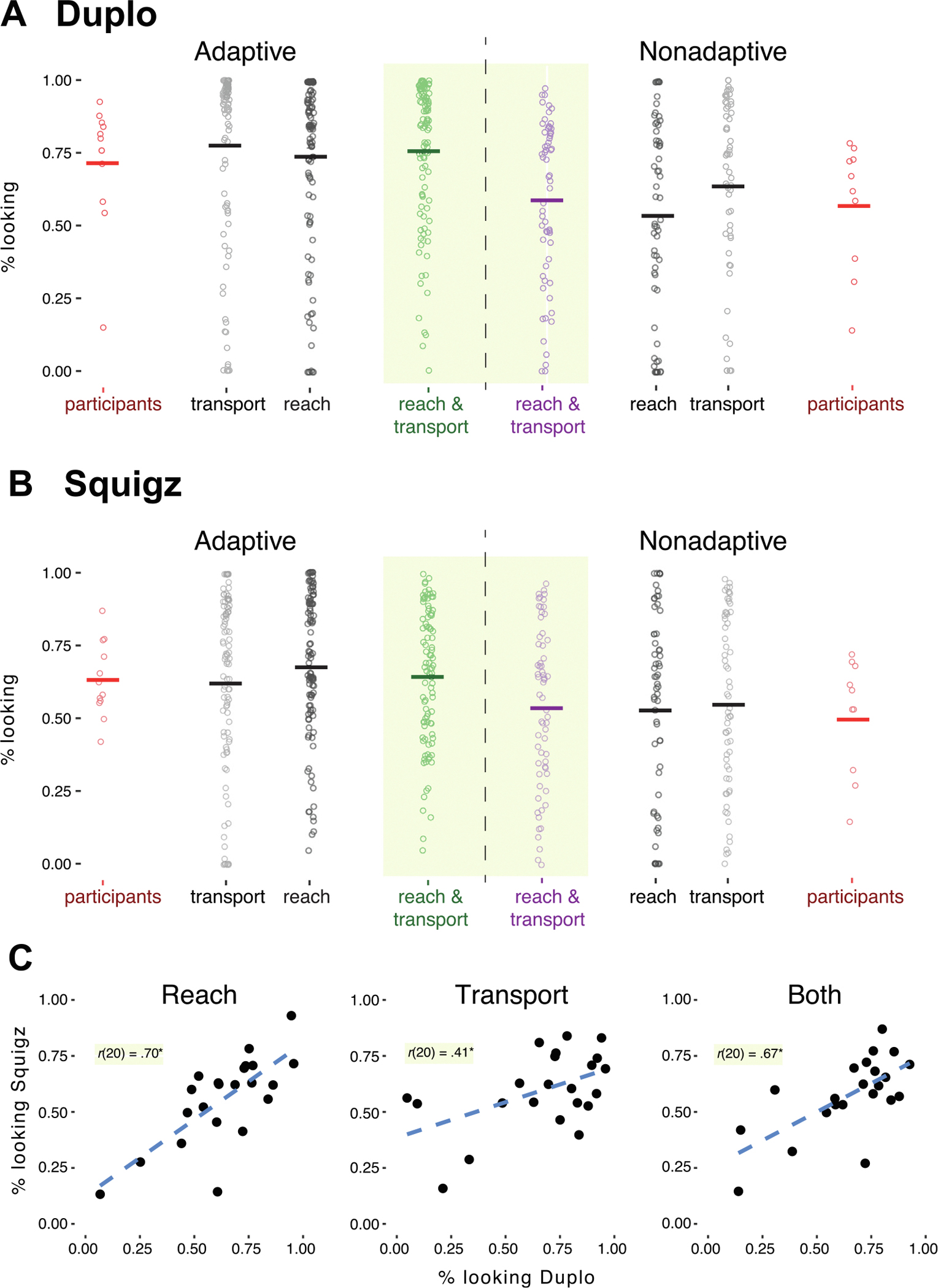
Looking in free-play tasks: Looking during free play with (A) Duplo bricks and looking during free play with (B) Squigz pieces. Differences between adaptive children (left panel) and nonadaptive children (right panel) in the proportion of time looking at the target object and construction in each free-play task are shown. Yellow areas with green and purple dots show the proportion of looking during reach and transport for adaptive and nonadaptive children, respectively. Each dot denotes a single trial. Dark gray dots show the proportion of looking only during reach, and light gray dots show the proportion of looking only during transport. Orange dots represent the average proportion of looking for individual children during reach and transport. (C) Correlations in looking between free play with Duplo blocks and Squigz pieces. The proportion of looking during reach (left), transport (middle), and both reach and transport (right) in the Duplo free-play task was correlated with the corresponding Squigz free-play task. Each dot represents a single child.

As predicted, children who planned more efficiently in the tool-use hammering task also planned more during free play with Duplo bricks. Adaptive children looked longer at the target Duplo brick and construction during combined reach and transport (*M* = 76% of reach and transport time, *SD* = 25) than nonadaptive children (*M* = 59%, *SD* = 28), *χ*^2^(,1) = 6.12, *p* = .01 (Fig. 2A, yellow panel). Analyzed separately for each phase, adaptive children looked longer during the reach, *χ*^2^(1) = 8.45, *p* = .004 (*M* = 74%, *SD* = 31), and marginally longer during transport, *χ*^2^(1) = 3.39, *p* = .07 (*M* = 77%, *SD* = 31), than nonadaptive children (*M*_reach_ = 54%, *SD* = 35; *M*_transport_ = 63%, *SD* = 32). Looking did not correlate with children’s age at either of the phases, *r*s < .13, *p*s > .58.

Results were similar for Squigz pieces (Fig. 2B, yellow panel). Adaptive children looked longer at the target Squigz piece and construction during reach and transport (*M* = 65% of reach and transport time, *SD* = 23) than nonadaptive children (*M* = 55%, *SD* = 28), *χ*^2^(1) = 5.06, *p* = .03. However, this difference was driven by looking during transport, *χ*^2^(1) = 6.81, *p* = .009 *M*_adaptive_ = 67%, *SD* = 26; *M*_nonadaptive_ = 56%, *SD* = 30), because looking did not differ between adaptive and nonadaptive children during the reach, *χ*^2^(1) = 2.66, *p* = .10 (*M*_adaptive_ = 62%, *SD* = 31; *M*_nonadaptive_ = 54%, *SD* = 34). Similar to the Duplo condition, looking was not correlated with children’s age at either of the phases, *r*s < .03, *p*s > .90.

Also as predicted, adaptive children looked more at the target object and construction during the reach and transport phases (separately or combined) when playing with the more familiar Duplo bricks than with the novel Squigz pieces, *χ*^2^s > 6.72, *p*s < .01 (Fig. 2A and 2B, green dots). However, nonadaptive children did not differ in their looking across tasks, *χ*^2^s < 2.84, *p*s > .09 (Fig. 2A and 2B, purple dots). Finally, as predicted, looking behavior was correlated across toys in both the reach and transport phases and when combining both together (Fig. 2C), *r*s > .41, *p*s < .05.

## Discussion

Visually guided planning of manual actions in 3- to 5-year-old children generalized from a structured tool-use task to unstructured free play with construction toys. Children who displayed adaptive planning during reach and transport when instructed to hammer a peg also displayed more adaptive planning during reach and transport in spontaneous free play with construction toys (Duplo bricks and Squigz pieces) compared with children who displayed nonadaptive planning in the structured hammering task. Specifically, during free play, adaptive planners looked more at the target objects and constructions during reach (prior to grasp) and during transport (prior to interlocking) than nonadaptive planners. In other words, adaptive planners generated visual information to guide their subsequent actions. Findings suggest that the development of planning is a domain-general process for manual actions and that visually guided planning provides the foundation for adaptive action in both multi-step and single-step tasks.

Reach, transport, and manipulation necessarily unfold in sequence. If looking occurs at the beginning of this real-time cascade, then visual information can smoothly and efficiently guide the subsequent actions with appropriate grips, efficient reach and transport trajectories, and so on (Ossmy et al., 2020, 2022). Without the initial look to jumpstart the planning cascade, the subsequent manual actions go awry. For fast actions like reaching for a handle, a Duplo brick, or a Squigz piece, children must plan to put their eyes on the relevant parts of the scene. Such “planning to plan” develops across early childhood (Jung et al., 2015; Keen, 2011; Lockman, 2008; Ornkloo & von Hofsten, 2007). Our findings indicate that children adept at multi-step planning, as evidenced by their performance in the hammering task, actively gather visual information in other manual tasks earlier than children who are not yet adept at multi-step planning of manual actions. Although children received no explicit instructions in the free-play tasks, and playing with the construction toys did not necessitate gathering of visual information during reach and transport, presumably the adaptive planners had learned that visual guidance is critical for planning actions efficiently.

Our study paves the way for a more naturalistic approach to research on action planning. Prior studies of planning during hammering, shape-sorter, spoon, and tower-building tasks entailed explicit instructions about what actions children should do (Chen et al., 2010; Jung et al., 2015, 2018; McCarty et al., 2001; Ossmy et al., 2020). Here, we demonstrated that children’s planning skills can be effectively studied in unstructured free play, not just in structured tasks. Structured laboratory tasks often impose artificial constraints that might not accurately reflect children’s everyday experiences (Dahl, 2017). In contrast, free play provides a rich context for observing natural behaviors and cognitive processes. Our study shows that complex planning behaviors, typically observed in structured tasks, are equally evident in more natural self-directed play scenarios.

The positive correlations for looking during planning across the two toys, Duplo bricks and Squigz pieces, provide further evidence for the generalizability of visually guided planning. That is, children develop perceptual strategies of where to look before they act, and these strategies are applied broadly across tasks and environments. Our findings illuminate how children adapt to the complexities of real-world situations (Chen et al., 2010; Keen et al., 2003, 2014; Paulus, 2016). Moreover, the correlation between the two toys during reach was stronger than during transport, suggesting that children’s visually guided planning during the first reach phase was more consistently generalized across different tasks than during the transport phase. This may indicate that the strategies children use to gather visual information before grasping an object are more stable and transferable across contexts, whereas the transport phase may require more task-specific adjustments. In other words, reaching for an object relies on more fundamental perceptual–motor skills that are applied broadly, whereas transporting an object is more influenced by the unique properties of each object or the specific demands of the task. Finally, our findings highlight the need for future studies on the development of planning across contexts. Longitudinal studies could provide deeper insights into how planning skills evolve, which factors contribute to generalizability of learning, and how the ability to generalize depends on children’s expertise or knowledge within a specific domain (Gobbo & Chi, 1986).

## Data Availability

The data is shared via Databrary and details appear in the manuscript
